# The Thai Internalized HIV-related Stigma Scale

**DOI:** 10.3389/fpsyg.2023.1134648

**Published:** 2023-03-15

**Authors:** Penpaktr Uthis, Sunisa Suktrakul, Rangsima Wiwatwongwana, Arunrat Tangmunkongvorakul, Patumrat Sripan, Kriengkrai Srithanaviboonchai

**Affiliations:** ^1^Faculty of Nursing, Chulalongkorn University, Bangkok, Thailand; ^2^Faculty of Social Sciences, Chiang Mai University, Chiang Mai, Thailand; ^3^Research Institute for Health Sciences, Chiang Mai University, Chiang Mai, Thailand; ^4^Faculty of Medicine, Chiang Mai University, Chiang Mai, Thailand

**Keywords:** HIV, people living with HIV, Thailand, internalized HIV-related stigma, stigma, scale development

## Abstract

**Introduction:**

Internalized stigma among people living with HIV has been linked to a range of negative consequences. The current study describes the development and validation of a contextually appropriate internalized HIV-related Stigma Scale for people living with HIV in Thailand.

**Methods:**

The study was carried out in two stages from 2018 to 2019: developing items based on the findings of focus group discussions and pilot testing the original list of items and validating the instrument. In the cross-sectional survey stage, a sample of 400 people living with HIV was used to validate the test items in accordance with their psychometric characteristics.

**Results:**

The study’s outcome was the 22-item Thai Internalized HIV-related Stigma Scale (Thai-IHSS). The exploratory factor analysis showed that the Thai-IHSS consisted of four components: negative thoughts toward self (5 items), anticipated negative thoughts (7 items), effects of negative thought toward self (6 items), and effects of negative thoughts toward family and access to healthcare services (4 items).

**Discussion:**

The Thai-IHSS had acceptable concurrent, convergent, and congruent validity according to the findings. Additionally, the 8-item Thai-IHSS brief, which included two items for each component, was detailed. The Thai-IHSS is valid and reliable for use in Thailand and other countries with comparable sociocultural environments.

## Introduction

HIV-related stigma refers to negative opinions of the illness and has been acknowledged as a potential issue ever since the beginning of the HIV epidemic (Stapleton, 1986; Elford, 1987). It is a significant issue for people living with HIV. HIV-related stigma restricts access to healthcare, which affects HIV treatment adherence (Katz et al., 2013; Sweeney and Vanable, 2016; Christopoulos et al., 2019; Camacho et al., 2020; Pearson et al., 2021). International organizations such as UNAIDS see HIV-related stigma as a significant barrier to putting an end to the HIV epidemic. Prevention of stigma and discrimination was recognized as a significant component of societal enablers which is essential to achieve the UNAIDS 2025 targets according to the latest modeling (Stover et al., 2021). Therefore, the alleviation of HIV-related stigma has been advocated for inclusion in national HIV programs (Grossman and Stangl, 2013).

Stigma associated with HIV can be categorized in a variety of ways. Earnshaw & Chaudoir classified HIV-related stigma as enacted (actual experience with prejudice or discrimination due to HIV), anticipated (the extent to which people living with HIV fear they will be discriminated as a result of having HIV), and internalized (endorsement of negative feelings and beliefs about HIV) (Earnshaw and Chaudoir, 2009). Each form of HIV-related stigma has distinct meanings and effects on people living with HIV health and quality of life.

Researching internalized HIV-related stigma (IHS) and developing interventions to lessen it is beneficial for a number of reasons. Unlike other types of stigma, IHS may be applicable to every people living with HIV. Some people living with HIV may not have revealed their HIV status, in which case they would worry less about enacted or anticipated stigma. For IHS, people living with HIV can also resolve the issue on their own without the need of assistance from others. IHS has also been linked to a number of adverse outcomes in people living with HIV. In a meta-analysis, it was found that IHS was associated with less social support, lower levels of access to and use of health and social services, greater rates of depression, and lower levels of adherence to antiretroviral drug regimens (Rueda et al., 2016). Poor medical adherence may also be explained by suboptimal people living with HIV care retention, which was partly driven on by IHS (Christopoulos et al., 2019; Pearson et al., 2021).

Researchers have tried to understand why IHS and poor health outcomes are related. Possible explanations include increased susceptibility to mental health issues, decreased self-efficacy, and worries about unintentional HIV status disclosure (Sweeney and Vanable, 2016). A meta-analysis also found that the causes of the linkage between IHS and unsatisfactory ART adherence were impairments of key psychological processes such as social support and adaptive coping (Katz et al., 2013). On the other hand, IHS could be exacerbated by unfavorable HIV-related health conditions. Poor health was identified to be a predictor of increased IHS in three cohort studies. In two longitudinal studies, lower depression scores and improvements in general mental health predicted reductions in IHS (Pantelic et al., 2015).

Several IHS measurement tools have been developed and are now available for use by researchers and public health personnel (Berger et al., 2001; Sayles et al., 2008; Kalichman et al., 2009; Phillips et al., 2011; Li et al., 2016; Ferguson et al., 2022). Some of the widely used scales are Berger’s HIV Stigma Scale (Berger et al., 2001), Kalichman’s Internalized AIDS-related Stigma Scale (Kalichman et al., 2009), and Sayles’ Internalized Stigma Scale (Sayles et al., 2008). Each nation has unique sociocultural contexts that may influence how people perceive internalized stigma. For Thailand, these include a strong sense of family connectedness and Theravada Buddhist principles. Due to the issue’s cultural specificity, a localized IHS scale is required in order to measure the problem accurately. Such a tool has not yet been created for Thailand and other Asian nations with comparable sociocultural circumstances.

Due to a major pandemic in the past and successful antiretroviral program, Thailand has the highest adult HIV prevalence in Southeast Asia, at 1.0% (UNAIDS, 2023c). Myanmar and Cambodia came in second and third, with 0.8% (UNAIDS, 2023b) and 0.6% (UNAIDS, 2023a), respectively. It is estimated that 440,000 people are now living with HIV in the country (MoP Health, 2023). Thailand has achieved several accomplishments in the past 10 years toward reducing HIV-related stigma. Among these were the development of standardized measurement tools and regular data collecting and reporting systems to track HIV-related stigma situations in health settings (Srithanaviboonchai et al., 2017). The country also created a training program to lessen HIV-related stigma in hospital settings as well as an IHS reduction program, both of which have been scaling up nationally (Srithanaviboonchai et al., 2017; Siraprapasiri et al., 2020).

There are little data available on the extent of internalized HIV stigma in Thailand. In a recent publication, 400 people living with HIV/AIDS in Bangkok and Chiang Mai had an overall internalized stigma score of 71 out of 100 (Thapinta et al., 2022). Internalized HIV stigma is a widely acknowledged issue, and Thailand should be no exception. Thai people living with HIV were also found to have significant rates of depression, which is associated with internalized stigma (Aurpibul et al., 2022). There is still a need for a standardized measurement of IHS given that it is essential for Thailand to be able to monitor this phenomenon at the individual, institutional, and country level.

The objective of this study was to develop a standardized measurement for IHS among Thai people living with HIV. This tool will be employed in both the evaluation of the ongoing IHS reduction program and the national HIV-related HIV stigma monitoring. This newly created measurement tool should also be beneficial to neighboring nations with comparable sociocultural contexts to Thailand, like Myanmar, Laos, and Cambodia.

## Materials and methods

The study was divided into two stages. For stage 1, the questionnaire items were developed based on the findings of in-depth interviews and pilot testing the original list of items and other instruments to be used in stage 2. For stage 2, the questionnaire items were validated in accordance with their psychometric characteristics.

### Stage 1: Development of questionnaire items

The first stage of this study divided into two steps, qualitative study and developing the instrument’s items.

#### Qualitative study

After reviewing pertinent literature including various types of stigma and their fundamental concepts as well as existing instruments, especially internalized stigma among people living with HIV, the research team created a set of semi-structured, open-ended questions for a qualitative study. Questions included basic demographics, life contexts before and after knowing the HIV status, perceptions and experiences of HIV-related stigma and discrimination, feeling about themselves, and coping mechanisms.

The qualitative study was conducted at antiretroviral clinics in Bangkok and Chiang Mai (northern Thailand), 20 people living with HIV who met the inclusion criteria were purposively recruited as key informants from each clinic. Inclusion criteria were known HIV positive for at least 1 year, 18 years old or older, and be able to communicate in Thai. To capture wide variety of experiences, thoughts, and meaningful on self-stigma related to HIV in the Thai context, equally number of five subgroups of people living with HIV were targeted and recruited at both Bangkok and Chiang Mai sites. These include general adult, people who used drugs, men who have sex with men, transgender women, and sex workers. Each group had four individuals for each site, making a total of 40 key informants.

Research team members who have extensive experiences in qualitative research conducted the in-depth interviews at the clinics where the key informants were receiving care in a confidential environment. The interviews were in Thai with voice recorded and all audio files were then transcribed verbatim into Thai. From content analysis, four higher level conceptual domains of the Thai self-stigma were identified: (1) perceived stigma before and after knowing HIV status; (2) anticipated stigma; (3) negative thoughts; and (4) experienced stigma.

#### Thai-IHSS development

In this step, a pool 57 items of the Thai-IHSS were initially drafted by research team members in accordant with the four domains identified in a qualitative study as well as literature reviewed of related studies, 13–15 items for each domain. The pilot testing of instruments was conducted by the first author (in Bangkok site) and corresponding author (in Chiang Mai site) using two focus group interviews with the same key informants from the qualitative study, ten key informants from each site. This was done to evaluate the newly created IHS items’ language, clarity, understandability, and acceptability. In light of participant comments, 15 items with ambiguous meaning and redundancy were then eliminated. A 42-item revised version of the Thai-IHSS was modified as a 4-point rating scale (strongly disagree, disagree, agree, and strongly agree) to capture feelings/emotions, perceptions/thoughts, and experiences of internalized HIV-related stigma. The higher scores indicated higher HIV-related stigma.

### Stage 2: Survey to validate the newly developed instruments

#### Study settings

Purposefully chosen study locations included two hospitals in Bangkok and two in Chiang Mai that provided HIV care to people living with HIV.

#### Study participants

The sample size of our study was set at 400, based on a general rule of thumb mentioned by Tabachnick and Fidell (1996, p. 640) that “it is comforting to have at least 300 cases for factor analysis” and the subjects-to-variables (STV) ratio of “a ten-to-one” that would be more acceptable for EFA suggested by Hair et al. (1995, p. 373). Therefore, our total study subject was large enough to facilitate the validation of the newly developed instrument. Inclusion criteria were known HIV positive for at least 1 year, 18 years old or older, and be able to read Thai.

#### Research instruments

In Stage 2, the research team developed a collection of questions that would be applied of the study which included the demographic questionnaire, the 42-item of newly developed Thai-IHSS scale, the 9-item of the Thai version of the PHQ-9, the 28-item Sayles’ Internalized Stigma Scale (Sayles et al., 2008), and the 6-item Kalichman’s Internalized AIDS-related Stigma Scale (Kalichman et al., 2009). Before being employed, the Internalized AIDS-related Stigma Scale and the Sayles’ Internalized Stigma Scale were translated into Thai and back into English upon the permission of the instrument’s owners. For its reliability, the entire questionnaire was pilot tested with 30 people living with HIV, 15 in Bangkok and 15 in Chiang Mai province with the Cronbach Alpha reliability, except the demographic questionnaire, of 0.92, 0.83, 0.89, and 0.73, respectively.

#### Data collection

Antiretroviral clinics that were scheduled at the participating hospitals were where the data were collected. Consecutive recruits were made until the clinic’s target sample size was reached. The questionnaire was filled out independently by participants at the clinics where they were receiving care.

#### Data analysis

Descriptive statistical analysis included frequency, percentage, mean, and standard deviation. Exploratory factor analysis, concurrent validity, convergent validity, congruent validity, and discriminant validity were conducted to validate the quality of the developed instrument. This study used EFA because the conceptual of self-stigma among people living with HIV in the Thai context is not clear, this method is usually adopted when developing a new scale because it helps to identify a set of latent constructs by determining the number and nature of common factors needed to account for the pattern of correlations among the measured variables (Fabrigar et al., 1999; Brown, 2015).

For EFA, as suggested by Watkins (2021), we started with checking required basic assumption tests and found that the Kaiser–Meyer–Olkin (KMO) was 0.899, indicating that our data gathered from cross-sectional study had good sampling adequacy (>0.5) which is suitable for EFA analysis, and the Bartlett’s test of Sphericity revealed the Chi-Square of 5,121.34 (Sig. <0.05), indicating that those 42 items had enough significant relationship to run a meaningful EFA. After explored those assumptions, we ran factor extraction analysis using a principal component method used eigenvalues greater than 1.0 along with the scree plot with squared multiple correlations as communalities to provide an indication of the number of underlying factors (Cattell, 1966). Then, an oblique factor rotation (PROMAX) was performed to allow for inter-factor correlations; in this step, 31 items with factor loadings greater than 0.55, a good cut-off suggested by Tabachnick and Fidell (2007), were remained and 12 were excluded. After that, the 30 remained items were evaluated whether they highly correlated with their hypothesized scales (corrected for item overlap) more than they did with other scales using multitrait scaling analysis (Hays and Hayashi, 1990), we then excluded another 5 items with lower item discrimination, and an additional 3 items that overlapped with items having higher item-scale correlations and better item discrimination. This resulted in final 22-items defining four multi-item scales suggested by the factor analysis.

### Ethical considerations

This study was approved by the Human Experimentation Committee at the Research Institute for Health Sciences, Chiang Mai University (Certificate number 18/2018 and 24/2019). Each participant gave their written informed consent prior to data collection. Each participant received 300 baht as compensation for their time. Participants who were identified to have major depressive disorder were referred to treatment that was covered by their individual health insurance plans.

## Results

### Characteristics of survey participants

The majority of people living with HIV in this study were male (55.3%). Ages ranged from 19 to 69 years old (median = 44). The most common marital status was single (39%), the most common education level was primary school (30%), and the most common employment status was general employment (36%) (Table 1).

**Table 1:** Survey (Stage 2) participant characteristics.

Characteristics	*n* (%)
Sex	
Male	221 (55.3)
Female	179 (44.7)
Age (Median, IQR)	44 (36.0–52.0)
Marital status	
Single	156 (39.0)
Married (living together)	152 (38.0)
Married (separated)	34 (8.5)
Divorced/Widowed	58 (13.5)
Religion	
Buddhism	379 (94.8)
Christianity	15 (3.8)
Islam	4 (1.0)
No religion	2 (0.4)
Education	
Never went to school	8 (1.8)
Primary school	120 (30.0)
Junior High School	73 (18.3)
Senior High School/Vocational/Certificate	117 (29.3)
Bachelor’s degree or higher	82 (20.5)
Occupation	
Individually owned business	82 (20.5)
General employment	145 (36.0)
Private company employee	71 (17.8)
Government official	32 (8.0)
State enterprise official	10 (2.5)
Household business	5 (1.4)
Agriculture	24 (6.0)
No job	31 (7.8)
Type of health coverage	
Universal health coverage (Gold card)	233 (58.2)
Social security	132 (33.0)
Government officer	29 (7.2)
Healthcare for migrant workers	1 (0.3%)
No health coverage	5 (1.3%)
Household income (Thai Baht)	
Yearly income (Median, IQR)	180,000 (106,800–300,000)
Perceived financial status	
Enough to save	45 (11.2)
Enough to spend	222 (55.5)
Not enough to spend	133 (33.3)
Total	400 (100.0)

### The Thai Internalized HIV-related Stigma Scale (Thai-IHSS)

**Figure 1:**
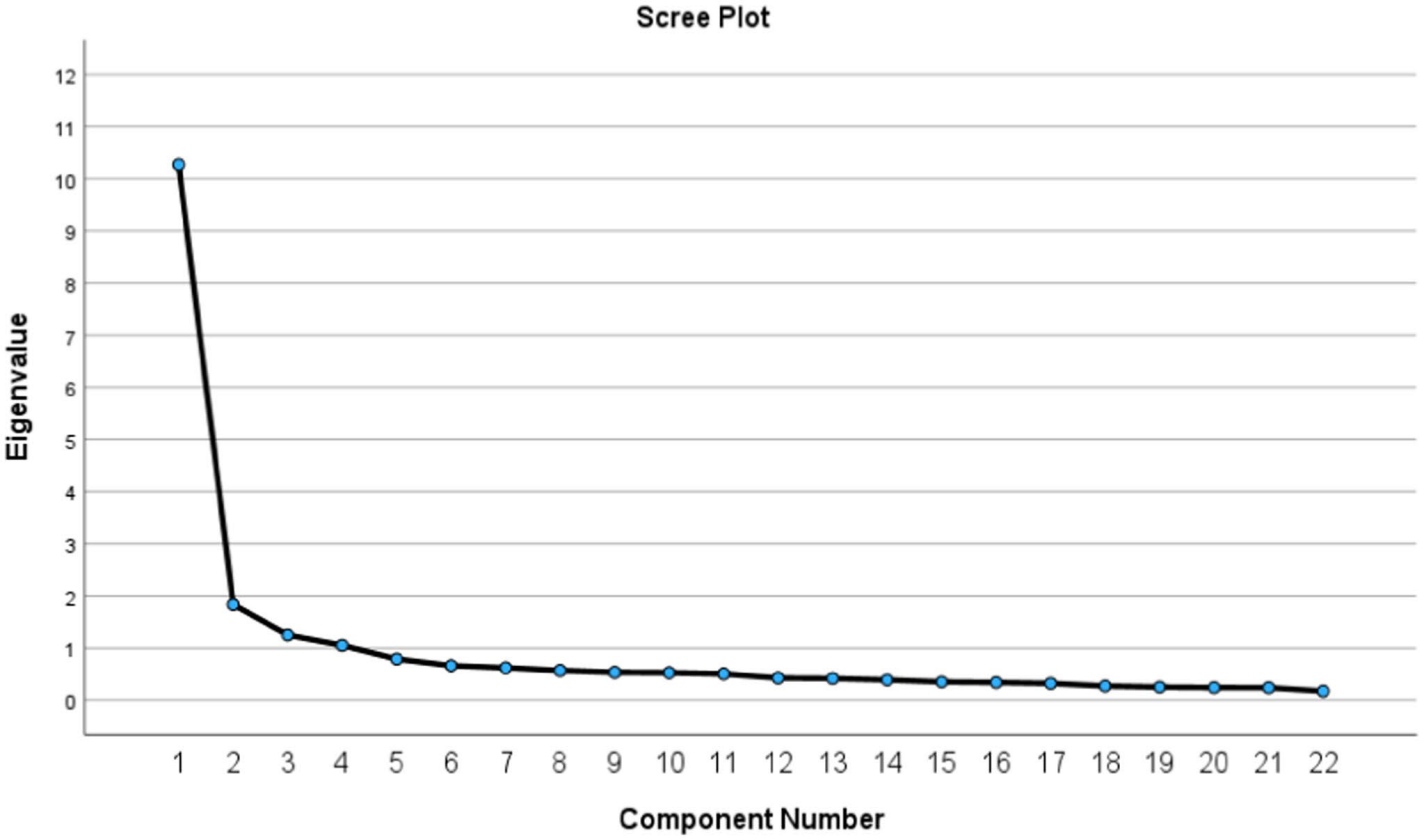
Scree plot of eigenvalues after EFA (*n* = 400).

From EFA, an oblique factor rotation (PROMAX) revealed that the test items with eigenvalues greater than one comprised four factors with the cumulative percentage of variance for all items of 65.03% (Table 2), which confirmed by the scree plot shown four existing underlying factors (Figure 1).

**Table 2:** Factor loading on the structural components of the 22-item Thai Internalized HIV-related Stigma Scale (Thai-IHSS).

Items	Factor			
	1	2	3	4
Factor 1 = Anticipated negative thoughts				
1. Others may end their relationships with me if they learn that I am infected with HIV.	0.802	0.412	0.588	0.518
2. Others would serve me right/aggravate me if they know that I am infected with HIV.	0.764	0.431	0.428	0.642
3. I feel bad for myself because I may transmit HIV to other people.	0.751	0.513	0.519	0.407
4. I fear that people will find me disgusting because I am infected with HIV.	0.737	0.340	0.610	0.454
5. I am afraid that I will be fired or not accepted for work because I am infected with HIV.	0.735	0.424	0.453	0.474
6. I feel being starred/gossiped from others because I am infected with HIV.	0.720	0.391	0.402	0.534
7. I feel that I am different from others because I am infected with HIV.	0.720	0.583	0.556	0.527
Factor 2 = Effects of negative thoughts toward self
8. I have the idea of dying because I am infected with HIV.	0.397	0.848	0.381	0.409
9. I want to hurt myself because I am infected with HIV.	0.355	0.834	0.398	0.415
10. I feel that everything I have done were wrong because I am infected with HIV.	0.539	0.834	0.360	0.524
11. I think I have no value because I am infected with HIV.	0.593	0.804	0.396	0.554
12. I feel discouraged/despaired because I am infected with HIV.	0.632	0.757	0.620	0.567
13. I think I do not have a future because I am infected with HIV.	0.625	0.697	0.255	0.686
Factor 3 = Negative thoughts toward self
14. I feel regret that I am infected with HIV.	0.445	0.383	0.805	0.406
15. I am ashamed that I am infected with HIV.	0.588	0.370	0.787	0.459
16. I feel angry with myself that I am infected with HIV.	0.490	0.475	0.774	0.388
17. I feel scared that I am infected with HIV.	0.570	0.534	0.746	0.466
18. I think that I am HIV-infected because of my bad karma.	0.510	0.519	0.553	0.511
Factor 4 = Effects of negative thoughts toward family and access to healthcare services				
19. I’m a bad person that makes my parents/family sad because I am infected with HIV.	0.479	0.540	0.432	0.843
20. I humiliate my family because I am infected with HIV.	0.563	0.516	0.451	0.803
21. I am afraid that my family will hate/abandon me if they know that I am infected with HIV.	0.472	0.361	0.466	0.735
22. I do not want to go to health services for fear that others will find out that I am infected with HIV.	0.581	0.561	0.205	0.655

A four-factor oblique rotation demonstrated that three of the factors consisted of items representing the four hypothesized domains. Items created from the hypothesized domain of “anticipated stigma” consistently loaded on factor 1, items from the domain “negative thoughts” consistently loaded on factor 3. Whereas items created from the domain “perceived stigma before and after knowing HIV status” collapsed with “experienced stigma,” loading on factor 2. Finally, a new domain (not hypothesized) emerged in factor 4, comprised items related to individual’s negative thoughts or concerns on HIV status toward family and access to healthcare services. Most of the items in the new domain indicated the family tie, which is a uniqueness of the Thai family such as the item “I’m a bad person that makes my parents/family sad because I am infected with HIV” and the item “I am afraid that my family will hate/abandon me if they know that I am infected with HIV.” There was an item “I do not want to go to health services for fear that others will find out that I am infected with HIV” that not related to family; however, due to its importance in the achievement goal of ending AIDS in Thailand and its factor loading that higher than 0.60, we decided to keep this item in the new domain. Therefore, in order to reflect the full range of items, we named this new domain as “effects of negative thoughts toward family and access to health care services.”

In order to have more meaningful and suitability for the Thai context, we then revised the name of each factors. Therefore, the final version of the Thai Internalized HIV-related Stigma Scale (Thai-IHSS) comprised 22 items with four factors named as the following. A list of how many items are included in each factor and the factor loading of each items are also shown in Table 2. Factor 1 is “Anticipated Negative thoughts” comprised 7 items with a range of 0.720–0.802 factor loading; Factor 2 is “Effects of Negative Thoughts Toward Self” comprised 6 items with a range of 0.697–0.848 factor loading; Factor 3 is “Negative Thoughts Toward Self” comprised 5 items with a range of 0.553–0.805 factor loading; and Factor 4: “Effects of negative thoughts toward family and access to health care services” comprised 4 items with a range of 0.655–0.843 factor loading. All items in each factor had factor loadings greater than a good cut-off 0.55 (Tabachnick and Fidell, 2007).

Table 3 illustrates the descriptive statistics, internal consistency reliability, and the Corrected Item-Total Correlation of the Thai-IHSS scale. Mean scores of the four subscales are 16.96, 11.03, 12.68, and 8.23 for subscale “Anticipated Negative thoughts,” “Effects of Negative Thoughts Toward Self,” “Negative Thoughts Toward Self,” and “Effects of negative thoughts toward family and access to health care services,” respectively. The four subscales and the Thai-IHSS’s overall scale have a normal distribution with the Skewness ranged from −0.08 to 0.29 and Kurtosis ranged from 0.12 to 0.53 which are acceptable values according to Brown (2006) (skewness fall between −3 and + 3, and kurtosis from a range of −10 to +10). In addition, the four subscales and the overall scale have a good reliability supported by the internal consistency reliability of 0.81–0.95 (Cortina, 1993) and the corrected item-total correlation of 0.721–0.767 which confirmed that each subscale had a good correlation with other subscales (Zijlmans et al., 2019). The scale also having good and significant intercorrelations among the four scales ranged between 0.606 and 0.715 (*p* < 0.01) (Table 4).

**Table 3:** Descriptive statistics and internal consistency (Cronbach Alpha) of the subscale and overall scale of the Thai Internalized HIV-related Stigma Scale (Thai-IHSS).

**Domain/subscale**	**No. of items**	**Mean score (Min-Max)**	**Median score**	**SD**	**Skewness**	**Kurtosis**	**Internal consistency** ^**a**^	**Corrected item-total correlation**
Anticipated negative thoughts	7	16.96 (7–28)	17	4.61	−0.08	0.12	0.90	0.767
Effects of negative thoughts toward self	6	11.03 (6–24)	12	3.53	0.12	1.55	0.90	0.724
Negative thoughts toward self	5	12.68 (5–20)	13	3.36	−0.11	0.16	0.84	0.752
Effects of negative thoughts toward family and access to healthcare services	4	8.23 (4–16)	8	2.63	0.29	0.24	0.81	0.721
Overall	22	48.90 (22–88)	49	12.18	0.16	0.53	0.95	N/A

a Cronbach’s Alpha reliability.

**Table 4:** Product–moment correlation among the Thai Internalized HIV-related Stigma Scale (Thai-IHSS).

**Scale**	**1**	**2**	**3**	**4**
1. Anticipated negative thoughts	–			
2. Effects of negative thoughts toward self	0.628**	-		
3. Negative thoughts toward self	0.606**	0.657**	-	
4. Effects of negative thoughts toward family and access to healthcare services	0.715**	0.640**	0.642**	-
Overall	0.864**	0.843**	0.802**	0.903**

** Pearson product–moment coefficients, *p* < 0.01.

To confirm the validity of the Thai-IHSS, we explored the instrument’s criterion-related validity by comparing mean scores of four subscales and total scale regarding HIV disclosed status and found that people living with HIV who disclosed their HIV status had significantly lower IHS scores for three subscales and total scale, except the subscale on effects of negative thoughts toward self, indicating that the Thai-IHSS has a good concurrent validity and will be able to predicts HIV disclosed status (Table 5). We tested for the instrument convergent validity by explored the relationship of the Thai-IHSS with similar construct instrument and found that it had a significant positive correlation with Sayles’ Internalized Stigma Scale (*r* = 0.556, *p* < 0.001) and Kalichman’s Internalized AIDS-related Stigma Scale (*r* = 0.564, *p* < 0.001), the Thai-IHSS also exhibited congruence validity, which was demonstrated by a significant positive association with the PHQ-9 scores (*r* = 0.453, *p* < 0.05) (Table 6).

**Table 5:** Product–moment correlation among the Thai Internalized HIV-related Stigma Scale (Thai-IHSS).

Thai-IHSS subscale	HIV discloser status	*N*	Mean	Std. Deviation	Std. Error mean	*t* ^a^	*p*-value
Anticipated negative thoughts	Not disclosed	117	18.2222	4.26314	0.39413	3.57	0.000
	Disclosed	283	16.4417	4.64857	0.27633		
Effects of negative thoughts toward self	Not disclosed	117	11.3675	3.38505	0.31295	1.23	0.220
	Disclosed	283	10.8905	3.59156	0.21350		
Negative thoughts toward self	Not disclosed	117	13.4530	3.29445	0.30457	3.01	0.003
	Disclosed	283	12.3534	3.33936	0.19850		
Effects of negative thoughts toward family and access to healthcare services	Not disclosed	117	9.0855	2.68319	0.24806	4.26	0.000
	Disclosed	283	7.8799	2.52920	0.15035		
Total Thai-IHSS (22 items)	Not disclosed	117	52.1282	11.76387	1.08757	3.46	0.001
	Disclosed	283	47.5654	12.11229	0.72000		

a df = 398 for all subscales and total scale.

**Table 6:** Pearson product–moment coefficients between the Thai-IHSS and other instruments.

Scale	*r*
Sayles’ Internalized Stigma Scale	0.556**
Kalichman’s Internalized AIDS-Related Stigma Scale	0.564**
PHQ-9	0.453*

*p < 0.05; **p < 0.001.

### The Thai Internalized HIV-related Stigma Scale brief (Thai-IHSS brief)

The discriminant power of the items, defined as the capacity of the items to differentiate between people living with HIV with high IHS scores (Quartile 75 and above) and those with low IHS scores (Quartile 25 and lower), was examined. Using the top two items for each component in terms of discriminant power, we created a list of the best eight items drawn from the four components that could be used as a brief version of the newly developed instrument in this study. Table 7 shows the 8-item Thai-IHSS brief with their discriminant power.

**Table 7:** The 8-item Thai-IHSS brief with their discriminant power.

Component	Thai-IHSS brief item	*t* *	*p* value
Negative thoughts toward self	I am ashamed that I am infected with HIV.	5.374	<0.001
	I think that I am HIV-infected because of my bad karma.	5.184	<0.001
Effects of negative thoughts toward self	I feel discouraged/despondent because I am infected with HIV.	5.335	<0.001
	I think I do not have a future because I am infected with HIV.	5.022	<0.001
Effects of negative thoughts toward family and access to healthcare services	I do not want to go to health services for fear that others will find out that I am infected with HIV.	4.966	<0.001
	I humiliate my family because I am infected with HIV.	4.299	<0.001
Anticipated negative thoughts	Others would think it serves me right if they know I am infected with HIV.	5.856	<0.001
	Others may end their relationships with me if they learn that I am infected with HIV.	5.037	<0.001

* *t*-test for equality of means.

## Discussion

In this study, we developed and validated a tool to measure IHS specifically for Thai people living with HIV. IHS, a kind of HIV-related stigma, is a significant problem for people living with HIV. The study used a qualitative study to raise a comprehensive list of items relevant to the Thai context. A cross-sectional study was conducted to validate the items’ psychometric properties and select appropriate items. The final product includes the Thai HIV-IHSS, which has 22 items, and the Thai HIV-IHSS brief, which has 8 elements. The two measures can be separated into four components, namely “negative thoughts toward self,” “effects of negative thoughts toward self,” “effects of negative thought toward family and access to health care services,” and “anticipated negative thoughts.”

The Thai-IHSS, the major outcome of the study, is a multidimensional measurement of IHS in people living with HIV. It can be classified into four scales based on the findings of the factor analysis. The advantage is that it allows the user the choice to view the outcomes individually for each component or collectively. The majority of the IHS scales for people living with HIV that are currently available lack this feature. The Sayles’ Internalized Stigma Scale (Sayles et al., 2008), a well-known IHS scale with this property, contains four components as well. However, the subscales of this measurement are different from ours namely “Stereotype,” “Disclosure concerns,” “Social relationships,” and “Self-acceptance.” Another IHS which has subscales is from China (Li et al., 2016). It comprises two subscales, “Being refused” and “Guilt.”

Three items on effects of negative thoughts toward family have emerged and are included in the Thai-IHSS. The relatively high number emphasizes how significant family ties are to Thai culture. We found the items related to family ties in only one other IHS scale. The 2 items are “I feel abandoned by family members because I have HIV” and “My family is comfortable talking about my HIV” in Sayles’ Internalized Stigma Scale (Sayles et al., 2008).

Among the measurement items that were chosen to be included in the Thai-IHSS and the Thai-IHSS brief, one item in particular stood out from the rest and is worth mentioning. It reads “I think that I am HIV-infected because of my bad karma.” The majority of Thais believe in karma, a Hindu and Buddhist concept which states that one’s actions influence the likelihood of future good and bad outcomes. According to this idea, the causal relationship between actions and results is vague or occurs across lengthy durations in the cycle of reincarnation (Larson, 2020). The only IHS measurement item in other scales which is related to the karma concept was found in the IHS scale from China (Li et al., 2016). It reads “I must have done something wrong to deserve getting HIV.” However, this is not specific for the curse from the past lives. It can refer to just the bad behaviors in this present lives. Having this item in the scales made them distinctive for the people living with HIV in Thailand and the neighboring nations who share the same belief. According to a recent study in Myanmar, the majority of people living with HIV participants believed that their HIV infection was caused by their past karma (Aung et al., 2022). Similar to Thailand, in Myanmar, the majority of people are Buddhist. The inclusion of this item in the measures has some potential benefits. The caregivers can remind people living with HIV who hold this idea that they can place the blame for having HIV on actions in a past life rather than their current one. It is worthwhile to investigate whether this could be effectively applied in the internalized stigma reduction intervention.

A number of IHS-specific interventions have been developed. In the US, a comprehensive strategy helped reduce ISH for those newly entering HIV care (Yigit et al., 2020). A study with women living with HIV found that IHS may be reduced by cognitive behavioral treatment that addresses issues such as helplessness, guilt, and anger (Tshabalala and Visser, 2011). According to a recently published review, structural interventions, the prevention of drug stockouts, social empowerment, and wealth creation may help reduce ISH among people living with HIV (Pantelic et al., 2019). However, so far, no intervention has been demonstrated to lower IHS for Thai people living with HIV. The Thai-IHSS and the upcoming survey results from this measurement will be useful information in designing an intervention that is specifically targeted at ISH in Thai people living with HIV.

The Stigma Index is a global study carried out by networks of people living with HIV. It is an essential tool for assessing HIV-related stigma, as well as IHS in particular. Thailand has just concluded collecting data for the Stigma Index 2.0, the current version of the study. IHS was measured in this study using Kalichman’s Internalized AIDS-related Stigma Scale. The International Partnership who oversee this project did not allow modification of the questionnaire, so it was not possible to switch the measurement to the Thai-IHSS. However, the results of the Stigma Index 2.0 can be compared to those of other surveys that used the Thai-IHSS. This newly created scale will also be incorporated into the current Thai national surveillance on HIV-related stigma in the healthcare settings. We can track changes in internalized HIV stigma over time since the survey will be conducted every 2 years.

The fact that this study offers scales that are among the first ISH measuring tools to have been validated for use with Thai people living with HIV is one of its strengths. The primary list of items was also created using the perspectives of a wide range of people living with HIV, including general adults, people who used drugs, men who have sex with men, transgender women, and sex workers. For the Thai-IHSS, the user has the flexibility of examining the results for each component separately or collectively thanks to the four subscales. The measures are also available in both the complete version (Thai-IHSS) and the short version (Thai-IHSS brief), giving users options. However, our research does have certain limitations. Participants of the quantitative survey were conveniently recruited from government HIV clinics. The sample may not adequately represent the Thai people living with HIV who were the most stigmatized and may be biased toward those with favorable experiences in HIV clinics. The Thai-IHSS brief, while more convenient to use than the Thai-IHSS, has not yet been fully tested for its psychometric properties.

In conclusion, the current study provides a new set of standardized measurements for IHS, an important issue within the context of HIV-related stigma. The study’s findings indicate that the Thai-IHSS had good psychometric qualities. The Thai-IHSS brief, a simplified version of the measurement, is also offered as an alternative. The scales appear to be a reliable and valid tool to measure IHS among Thai people living with HIV and people living with HIV in other countries with similar sociocultural settings. The study’s findings might be valuable for future research and clinical assessment.

## Data availability statement

The raw data supporting the conclusions of this article will be made available by the authors, without undue reservation.

## Ethics statement

The studies involving human participants were reviewed and approved by the Human Experimentation Committee, Research Institute for Health Sciences, Chiang Mai University. The patients/participants provided their written informed consent to participate in this study.

## Author contributions

PU and KS conceived the study. PU, SS, AT, RW, and KS collected the data. PU, AT, PS, and KS contributed to data analysis and interpretation. PU and KS drafted the manuscript. All authors contributed to the article and approved the submitted version of the manuscript.

## Funding

This study was supported by internal Chiang Mai University Research Funds. The funder played no role in study design or interpretation.

## Conflict of interest

The authors declare that the research was conducted in the absence of any commercial or financial relationships that could be construed as a potential conflict of interest.

## Publisher’s note

All claims expressed in this article are solely those of the authors and do not necessarily represent those of their affiliated organizations, or those of the publisher, the editors and the reviewers. Any product that may be evaluated in this article, or claim that may be made by its manufacturer, is not guaranteed or endorsed by the publisher.
