# Supplementary material for: The Thai Internalized HIV-related Stigma Scale
Source: Front Psychol. 2023 Mar 15;14:1134648. doi: 10.3389/fpsyg.2023.1134648 (PMC10062318; doi:10.3389/fpsyg.2023.1134648)
Supplement: Supplementary file 1 [file Table_1.docx]

Supplementary table: Thai translated questionnaire items

| Factor 1: Anticipated negative thoughts  องค์ประกอบ 1: ความคิดล่วงหน้าในด้านลบ |
| --- |
| 1. Others may end their relationships with me if they learn that I am infected with HIV.   คนอื่นอาจจะเลิกคบกับฉัน ถ้ารู้ว่าฉันติดเชื้อเอชไอวี |
| 1. Others would think it serves me right if they know I am infected with HIV.   คนอื่นจะสมน้ำหน้า/ซ้ำเติม ถ้ารู้ว่าฉันติดเชื้อเอชไอวี |
| 1. I feel bad for myself because I may transmit HIV to other people.   ฉันรู้สึกไม่ดีกับตัวเอง เพราะอาจแพร่เชื้อเอชไอวีให้คนอื่น |
| 1. I fear that people will find me disgusting because I am infected with HIV.   ฉันกลัวคนรังเกียจเนื่องจากฉันติดเชื้อเอชไอวี |
| 1. I am afraid that I will be fired or not accepted for work because I am infected with HIV.   ฉันกลัวว่าฉันจะถูกไล่ออกจากงานหรือไม่มีงาน เนื่องจากฉันติดเชื้อเอชไอวี |
| 1. I feel being starred/gossiped from others because I am infected with HIV.   ฉันรู้สึกว่าถูกจ้องมอง/นินทา เนื่องจากฉันติดเชื้อเอชไอวี |
| 1. I feel that I am different from others because I am infected with HIV.   ฉันรู้สึกว่าตนเองแตกต่างจากคนอื่น เนื่องจากฉันติดเชื้อเอชไอวี |
| Factor 2: Effects of negative thoughts toward self  องค์ประกอบ 2: ความคิดด้านลบที่ส่งผลต่อตนเอง |
| 1. I have the idea of dying because I am infected with HIV.   ฉันมีความคิดอยากตายเนื่องจากฉันติดเชื้อเอชไอวี |
| 1. I want to hurt myself because I am infected with HIV.   ฉันอยากทำร้ายตนเองเนื่องจากฉันติดเชื้อเอชไอวี |
| 1. I feel that everything I have done were wrong because I am infected with HIV.   ฉันรู้สึกว่าทำอะไรก็ผิดไปหมดเนื่องจากติดเชื้อเอชไอวี |
| 1. I think I have no value because I am infected with HIV.   ฉันคิดว่าตนเองไม่มีคุณค่าเนื่องจากติดเชื้อเอชไอวี |
| 1. I feel discouraged/despaired because I am infected with HIV.   ฉันรู้สึกท้อแท้/สิ้นหวัง เนื่องจากฉันติดเชื้อเอชไอวี |
| 1. I think I don't have a future because I am infected with HIV.   ฉันคิดว่าฉันไม่มีอนาคตแล้ว เนื่องจากฉันเป็นผู้ติดเชื้อเอชไอวี |
| Factor 3: Negative thoughts toward self  องค์ประกอบ 3: ความคิดด้านลบต่อตนเอง |
| 1. I feel regret that I am infected with HIV.   ฉันรู้สึกเสียใจที่ฉันติดเชื้อเอชไอวี |
| 1. I am ashamed that I am infected with HIV.   ฉันรู้สึกอายที่ฉันติดเชื้อเอชไอวี |
| 1. I feel angry with myself that I am infected with HIV.   ฉันรู้สึกโกรธตัวเองที่ฉันติดเชื้อเอชไอวี |
| 1. I feel scared that I am infected with HIV.   ฉันรู้สึกกลัวที่เป็นผู้ติดเชื้อเอชไอวี |
| 1. I think that I am HIV-infected because of my bad karma.   ฉันรู้สึกว่าเป็นเวรเป็นกรรมที่ฉันติดเชื้อเอชไอวี |
| Factor 4: Effects of negative thoughts toward family and access to health care services  องค์ประกอบ 4: ความคิดด้านลบเกี่ยวกับครอบครัวและการรับบริการสุขภาพ |
| 1. I'm a bad person that makes my parents/family sad because I am infected with HIV.   ฉันเป็นคนไม่ดีที่ทำให้พ่อแม่/คนในครอบครัวเสียใจ เนื่องจากติดเชื้อเอชไอวี |
| 1. I humiliate my family because I am infected with HIV.   ฉันทำให้ครอบครัวอับอายเนื่องจากติดเชื้อเอชไอวี |
| 1. I am afraid that my family will hate/abandon me if they know that I am infected with HIV.   ฉันกลัวว่าครอบครัวจะรังเกียจ/ทอดทิ้ง ถ้ารู้ว่าฉันติดเชื้อเอชไอวี |
| 1. I don't want to go to health services for fear that others will find out that I am infected with HIV.   ฉันไม่อยากไปรับบริการสุขภาพ เพราะกลัวคนอื่นจะรู้ว่าฉันติดเชื้อเอชไอวี |
